# Inactivation of the phosphoglucomutase gene *pgm* in *Corynebacterium glutamicum* affects cell shape and glycogen metabolism

**DOI:** 10.1042/BSR20130076

**Published:** 2013-08-23

**Authors:** Gerd M. Seibold, Bernhard J. Eikmanns

**Affiliations:** *Institute of Biochemistry, University of Cologne, D-50674 Cologne, Germany; †Institute of Microbiology and Biotechnology, University of Ulm, D-89069 Ulm, Germany.

**Keywords:** α-glucose-1-phosphate, carbon capacitor, cell morphology, *Corynebacterium glutamicum*, glycogen metabolism, maltose metabolism, *pgm*, phosphoglucomutase, CBB, Coomassie Brilliant Blue, dw, dry weight, glc-1-P, α-glucose-1-phosphate, glc-6-P, glucose-6-phosphate, IPTG, isopropyl β-d-galactopyranoside, MALDI–TOF, Matrix-assisted laser desorption ionization–time of flight, MalP, maltodextrinphosphorylase, ORF, open reading frame, Pgm, phosphoglucomutase, WT, wild-type

## Abstract

In *Corynebacterium glutamicum* formation of glc-1-P (α-glucose-1-phosphate) from glc-6-P (glucose-6-phosphate) by α-Pgm (phosphoglucomutase) is supposed to be crucial for synthesis of glycogen and the cell wall precursors trehalose and rhamnose. Furthermore, Pgm is probably necessary for glycogen degradation and maltose utilization as glucan phosphorylases of both pathways form glc-1-P. We here show that *C. glutamicum* possesses at least two Pgm isoenzymes, the *cg2800* (*pgm*) encoded enzyme contributing most to total Pgm activity. By inactivation of *pgm* we created *C. glutamicum* IMpgm showing only about 12% Pgm activity when compared to the parental strain. We characterized both strains during cultivation with either glucose or maltose as substrate and observed that (i) the glc-1-P content in the WT (wild-type) and the mutant remained constant independent of the carbon source used, (ii) the glycogen levels in the *pgm* mutant were lower during growth on glucose and higher during growth on maltose, and (iii) the morphology of the mutant was altered with maltose as a substrate. We conclude that *C. glutamicum* employs glycogen as carbon capacitor to perform glc-1-P homeostasis in the exponential growth phase and is therefore able to counteract limited Pgm activity for both anabolic and catabolic metabolic pathways.

## INTRODUCTION

The non-sporulating, Gram-positive, rod-shaped actinomycete *Corynebacterium glutamicum* is employed in the industrial-scale production of amino acids [1]. Moreover, this non-pathogenic bacterium is widely accepted as a useful model to depict metabolism and cell wall biogenesis of Corynebacterineae including the pathogenic *Mycobacterium tuberculosis* [2–4]. *C. glutamicum* utilizes various substrates, including sugars, organic acids and alcohols for growth and amino acid production [5–7]. In the course of cultivation in media containing sugars as carbon and energy source or when phosphate is limiting in the culture broth, *C. glutamicum* transiently accumulates large amounts of glycogen [8,9]. This transient accumulation of glycogen in *C. glutamicum* is different from the situation in other organisms [10–12] as the accumulated glycogen is already degraded in *C. glutamicum* in the course of the late exponential growth phase before the substrate is consumed and the stationary growth phase begins [8,13]. As generally described for bacteria [10,12], glycogen synthesis is catalysed in *C. glutamicum* by the consecutive action of the enzymes ADP-glucose pyrophosphorylase GlgC, glycogen synthase GlgA and the glycogen branching enzyme GlgB [8,14,15]. Besides, genes for the alternative α-glucan synthesis pathway via GlgE, recently identified in *Mycobacterium* and *Streptomyces* species [11,16], are also present in the *C. glutamicum* genome [17,18]. However, the contribution of the GlgE pathway to glycogen synthesis in *C. glutamicum* seems negligible, because inactivation of *glgC* as well as *glgA* abolished glycogen synthesis in *C. glutamicum* [8,15]. The substrate for ADP-glucose formation by GlgC, glc-1-P (α-glucose-1-phosphate) is generally formed from glc-6-P (glucose-6-phosphate) by α-Pgm (phosphoglucomutase) [19–21]. The Pgm reaction is reversible and in the direction of glc-6-P formation represents the last step of *C. glutamicum* glycogen degradation [13,22]. In addition, the Pgm reaction links the maltose and maltodextrin utilization pathways to the glycolysis, as in the course of these pathways also glc-1-P is formed [13,23,24].

In bacteria such as *Escherichia coli*, *Bacillus subtilis* and *Streptococcus gordonii* the inactivation of genes encoding Pgm enzymes brought about changes of the cell shape and size [25–28]. These morphological changes are probably entailed by the limited intracellular glc-1-P availability, as glucose-1-P functions as a precursor of various cell wall components in aforementioned bacteria. Analysis of the metabolic pathways deduced from the genome sequence of *C. glutamicum* [18] indicates that the Pgm catalysed formation of glc-1-P is also required for synthesis of the nucleotide sugars dTDP-glucose and UPD-glucose, two central intermediates in the synthesis rhamnose and trehalose, which both are essential cell wall constituents of Corynebacterineae [29–31].

Despite its central role in catabolism and anabolism, Pgm so far has only scarcely been studied in *C. glutamicum*. Pgm activity was shown to be present in cell extracts of *C. glutamicum* [13] and the ORF (open reading frame) *cg2800* has been annotated as Pgm gene *pgm* based on sequence comparisons [18]. We recently found *pgm* to be up-regulated in response to phosphate limitation [9], however, the gene product of *pgm* has not been analysed and its role for both the transient accumulation of glycogen and the metabolization of maltose has not yet been studied. In addition, the levels of the central intermediate glc-1-P so far have not been analysed in *C. glutamicum*. We here characterize Pgm activity in *C. glutamicum*, identify the *pgm* encoded isoenzyme as main Pgm and study the consequences of *pgm* inactivation in *C. glutamicum* on growth, the intracellular glc-1-P and glycogen levels, and morphology in the course of cultivations when Pgm is supposed to be required either for anabolism or catabolism.

## EXPERIMENTAL

### Bacterial strains, media and growth conditions

The bacteria used in this study were *E. coli* DH5α [32] and *C. glutamicum* WT (wild-type) (strain ATCC13032; American Type Culture Collection). *E. coli* and all pre-cultures of *C. glutamicum* were grown aerobically in TY complex medium [1.6% (w/v) tryptone/1% (w/v) yeast extract/0.5% NaCl] [33] at 37 and 30°C, respectively, as 50 ml cultures in 500 ml baffled Erlenmeyer flasks on a rotary shaker at 120 rev./min. For the main cultures of *C. glutamicum*, cells of an overnight pre-culture were washed twice with 0.9% (w/v) NaCl and then inoculated into TY medium or CgC minimal medium [34] containing the carbon sources indicated in the text. When appropriate, kanamycin (25 μg/ml) or chloramphenicol (10 μg/ml) was added to the media. Growth of *C. glutamicum* was followed by measuring the absorbance at 600 nm (*A*_600 nm_).

### Analysis of cytoplasmic glycogen and glc-1-P levels

For enzymatic analysis of intracellular glycogen, 5 ml samples of respective cultures were harvested, cell extracts were prepared and glycogen content was determined with amyloglucosidase as described previously [8]. Rapid sampling, inactivation of metabolism and separation of intracellular and extracellular fluids for the determination of intracellular concentrations of glc-1-P and trehalose were achieved by using silicon oil centrifugation with perchloric acid in the bottom layer [9,35]. Samples were neutralized with 25 μl of 1 M KOH, 5 M TEA (triethanolamine). The potassium perchlorate thereby generated was precipitated by incubation for 30 min at 4°C followed by centrifugation (5 min, 20000 ***g***, 4°C). The supernatant was transferred to a new vial and lyophilized for at least 1 day. The dried cell extracts were treated at 30°C for 90 min with 35 μl methoxamine hydrochloride in pyridine (20 mg/ml) and subsequently trimethylsilated with 65 μl MSTFA [*N*-methyl-*N*-(trimethylsilyl)triflouroactimide] for 1 h at 65°C. The concentrations of derivatized glc-1-P were determined by GLC using the TraceGC system (Thermo Finnigan) and a FS supreme 5 column (CS-Chromatographie). After injection of 0.3 μl (split ratio 1:25), separation was achieved under nitrogen gas flow (flow rate 1 ml/min) using the following time program: 2 min at 60°C, temperature gradient of 30°C/min to 140°C, temperature gradient of 2°C/min to 175°C, temperature gradient of 30°C/min to 320°C, 5 min at 320°. Column effluents were monitored by FID (flame ionization detection) at 300°C, *myo*-inositol was used as an internal standard and glc-1-P and glc-6-P (all purchased from Sigma-Aldrich) as external standards.

### DNA preparation, transformation and manipulations

Standard procedures were employed for plasmid isolation, for cloning and transformation of *E. coli* DH5α, as well as for electrophoresis [33]. *C. glutamicum* chromosomal DNA was isolated according to Eikmanns et al. [36]. Transformation of *C. glutamicum* was performed by electroporation using the methods of Tauch et al. [37]. PCR experiments were performed in a FlexCycler (AnalytikJena). Oligonucleotides were obtained from Eurofins MWG Operon. Cycling times and temperatures were chosen according to fragment length and primer constitution. PCR products were separated on agarose gels and purified using the Nucleospin extract II kit (Macherey & Nagel).

### Inactivation and homologous overexpression of *pgm* in *C. glutamicum*

Inactivation of the chromosomal *pgm* gene (orf*cg2800*) in *C. glutamicum* was performed essentially as described for the inactivation of the *glgB* gene [14], using the plasmid pDrive-IMpgm. This plasmid was constructed by PCR-amplification of a DNA fragment covering nucleotides 465–1056 of the annotated *pgm* gene, using primers pgm-IM-for (5′-CCACAACCCTCCTCGTGATG-3′) and pgm-IM-rev (5′-GGTATCTGCGGACCAACCTG-3′). The 592 bp PCR product was directly cloned into the TA-cloning vector pDrive (Qiagen) according to the manufacturer's instructions and the resulting vector pDrive-IMpgm transformed into *E. coli* DH5α. After isolation of the recombinant plasmid, it was electroporated into *C. glutamicum* WT. Integration of pDrive-IMpgm at the genomic *pgm* locus in *C. glutamicum* and thus inactivation of the *pgm* gene was confirmed by PCR using primers Pgm-full-rev (5′-GACACGTCCACTAGTTG-3′) and T7 (5′-TAATACGACTCACTATAGGG-3′) resulting in a specific 1358 bp product for *C. glutamicum* IMpgm. For homologous overexpression of *pgm*, it was amplified from genomic DNA of *C. glutamicum* using primers pgm-OE-for (5′-GGATCCTGTTAAGCCACCCTACTC-3′) and pgm-OE-rev (5′-GGTACCTGACACGTCCACTAGTTG-3′). The 1859 bp PCR product was cloned using the primer-generated BamHI and KpnI restriction sites into the expression vector pXMJ19 [38]. The constructed vector pXMJ19-pgm allows the IPTG (isopropyl β-d-galactopyranoside)-inducible expression of *pgm* in *C. glutamicum*.

### Enzyme assay and protein analysis

Pgm activity was measured in a coupled reaction with glc-6-P dehydrogenase essentially as recently described [13]. The reaction mixture contained 100 mM Tris/HCl (pH 7.4), 10 mM MgCl_2_, 1 mM NADP, 2 units of glc-6-P dehydrogenase (Roche Diagnostics) and in standard assays 5 mM glc-1-P. For determination of *K*_M_ values, glc-1-P concentrations were varied from 0.05 to 20 mM. Native PAGE and Pgm activity staining were performed essentially as described [39,40]. The gels were incubated in Pgm staining buffer (50 mM HEPES/NaOH, pH 7.5, 3.3 mM MgCl_2_ and 0.9 mM EDTA) for 10 min. Afterwards, the gels were incubated at 30°C for 1 h in the dark in Pgm staining solution [0.85 mM NADP, 3 mM glc-1-P, 250 μM NBT (Nitro Blue Tetrazolium), 33 μM phenazinemethosulfate and glc-6-P dehydrogenase (1 unit/ml) solved in Pgm staining buffer]. The reaction was stopped by incubation of the gel in 10% (v/v) acetic acid. Protein concentrations were determined using the Roti-Nanoquant kit (Roth) with BSA as the standard. SDS/PAGE was performed according to Laemmli [41].

### Purification of Pgm

For purification of the Pgm isoenzyme from cell extract of *C. glutamicum* WT, cells were grown at an absorbance at 600 nm of approx. 15 in 5 litres of CgC minimal medium with 2% (w/v) maltose as carbon source in a 10-litre Braun Biostat B fermentation system, washed twice in 50 ml buffer A (0.02 M sodium phosphate buffer, pH 6.8), resuspended in 50 ml of the same buffer and disrupted mechanically with a French pressure cell (SLM Aminco) at 1800 lbs/in^2^ (40 K cell) five times with intermittent cooling on ice. After removal of cellular debris by centrifugation (Eppendorf 5804 R centrifuge; 20000 ***g***; 4°C, 20 min), the supernatant was centrifuged at 60000***g***, 4°C, 1.5 h (Beckmann Optima L90-K ultracentrifuge) to remove the membrane fraction. The cytosolic fraction was diluted 1:5 with buffer A and applied to a HiPrep 16/10 Q FF column (GE Healthcare), equilibrated with buffer A for ion exchange chromatography. Absorbed proteins were eluted with a stepwise gradient consisting of 0.2, 0.3, 0.4 and 1 M NaCl. The fractions with maximal Pgm activity collected from three runs at 0.4 M NaCl were diluted 1:10 in buffer A containing 1 M (NH_4_)_2_SO_4_ and then applied to a HiPrep 16/10 Phenyl FF column (GE Healthcare), equilibrated in the same buffer for hydrophobic interaction chromatography. Absorbed proteins were eluted using a 500 ml linear gradient consisting of 1 M to 0 M (NH_4_)_2_SO_4_, fractions with maximal Pgm activity were collected between 0.2 M and 0.1 M (NH_4_)_2_SO_4_. After the exchange of the buffer of these fractions to 0.02 MES-HCl, pH 5.8 (buffer B) by using a HiPrep 26/10 desalting column (GE Healthcare) these fractions were applied to a Tricorn Mono Q 5/50 GL column (GE Healthcare), equilibrated with buffer B for ion exchange chromatography. Absorbed proteins were eluted using a 20 ml linear gradient consisting of 0 M to 1 M NaCl. For further purification, the fraction with maximum Pgm activity collected between 0.38 M and 0.47 M NaCl was applied to a HiLoad XK16/60 Superdex 200 PrepGrade gel filtration column (GE Healthcare), equilibrated with GF buffer (0.02 M sodium phosphate buffer and 0.15 M NaCl, pH 7.4). Elution was performed with a constant flow of 0.5 ml/min. The fractions were collected and screened by activity analysis. For molecular mass determination, the retention volume of Pgm was compared with that of standard proteins (LMW and HMW calibration kits, GE Healthcare). MALDI–TOF (matrix-assisted laser desorption ionization–time of flight) analysis of protein bands cut out of colloidal CBB (Coomassie Brilliant Blue)-stained gels was performed at IBG-1, Forschungszentrum Jülich, with a Voyager-DE STR biospectrometry workstation (Applied Biosystems) essentially as described [42].

### Microscopic imaging

Phase-contrast and fluorescence microscopy were performed as described [13] with 2 μl culture samples placed on microscope slides coated with an agarose (1%) layer and covered by a coverslip using a Zeiss Axio Imager M1 microscope system. Viability staining using the Live/Dead BacLight Bacterial Viability Kit (Molecular Probes) was performed as described by Seibold et al. [13]. DNA staining with Hoechst (Sigma-Aldrich) and membrane staining with Nile-Red (Molecular Probes) were performed as described by Donovan et al. [43].

### Computational analysis

Databank searches were carried out by using BLAST [44] and the KEGG (Kyoto Encyclopedia of Genes and Genomes; http://www.genome.ad.jp/) database [45]. The UniProt accession numbers for protein sequences and the corresponding ORFs annotated as Pgm and/or phosphomannomutase are *C. glutamicum* Q6M788*–cg0788* (*pmmB*), Q8NSD0*–cg0854* (*manB*), Q8NMN0*–cg2800* (*pgm*); *E. coli* P36938*–b0688* (*pgm*); *B. subtilis* P18159*–bsu09310* (*pgcA*); *Pseudomonas aeruginosa* P26276*–pa5322* (*algC*).

## RESULTS

### Pgm activity and glycogen content in *C. glutamicum*

**Figure 1:**
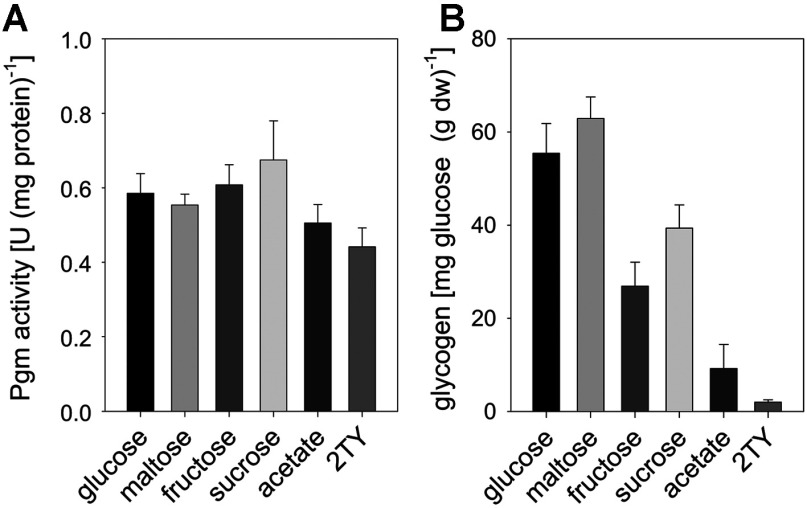
Specific Pgm activity (A) and glycogen content; (B) *C. glutamicum* WT cells sampled in the mid-exponential growth phase from cultivations in minimal medium with various carbon sources or TY complex medium Data represent mean values of three independent determinations from at least three independent cultivations.

As Pgm probably acts both as anabolic (e.g. for glycogen synthesis) and catabolic enzyme (e.g. for maltose utilization and glycogen degradation), we analysed Pgm activity and glycogen content in samples from the early exponential growth phase of *C. glutamicum* WT cultivations in minimal medium with various carbon sources and from cultivations in TY complex medium. As shown in Figure 1(A), all samples from the different cultivations contained relatively high and similar specific Pgm activities between 0.44±0.05 units/mg protein (TY medium) and 0.67±11 units/mg protein (minimal medium with sucrose). In contrast, the glycogen content varied significantly in dependence of the media used (Figure 1B). The highest glycogen contents were observed for cultivations with glucose or maltose as carbon source [55.4±6.3 and 62.9±4.6 mg glucose equivalents/g dw(dry weight), respectively], whereas only minor levels of glycogen were present in cells cultivated with acetate or in complex medium (9.2±5.1 and 1.4±0.2 mg glucose equivalents/g dw, respectively). Thus, there was no correlation between the glycogen content and the Pgm activities. Microscopic analysis revealed no changes of cell morphology of the *C. glutamicum* WT cells in the course of these cultivations in minimal medium with various carbon sources. Taken together, these results suggest that Pgm is constitutively present in *C. glutamicum* WT cells and probably not the limiting step for both glycogen metabolism and synthesis of cell wall components.

### Identification of the main Pgm isoenzyme of *C. glutamicum*

**Figure 2:**
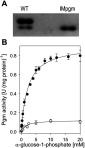
Pgm isoenzymes of *C. glutamicum* Cell extracts of *C. glutamicum* WT and *C. glutamicum* IMpgm were analysed by native PAGE followed by enzyme activity staining (**A**). Total Pgm activity in cell extracts of *C. glutamicum* WT (filled circles) and *C. glutamicum* IMpgm (open diamonds) at different concentrations (0.05–20 mM) of glc-1-P (**B**). Data represent mean values of three independent determinations from two independent cultivations and were fitted according to the Michaelis–Menten equation.

**Figure 3:**
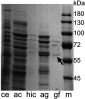
Purification of the main Pgm isoenzyme of *C. glutamicum*, SDS/PAGE analysis of each step of the purification procedure: ce (crude extract), aec (anion exchange chromatography step wise elution), hic (hydrophobic interaction chromatography), aeg (anion exchange chromatography with gradient), gf (gel filtration) and PAGE ruler prestained protein ladder (m, MBI Fermentas) containing proteins of indicated molecular mass The arrow points at the band, which was assigned to *pgm*.

*C. glutamicum* cell extracts were analysed by native PAGE followed by Pgm activity staining. As shown in Figure 2(A), C*. glutamicum* WT possesses at least two enzymes with Pgm activity. In fact, the genome sequence of *C. glutamicum* harbours three ORFs, whose deduced amino acid sequences share significant identities with well characterized proteins with Pgm activity from other bacteria: the protein encoded by *cg0788* possesses 29% identity to the Pgm PcgA of *B. subtilis* [27], the protein encoded by *cg0854* possesses 37% identical amino acids to the phosphomannomutase/Pgm AlgC of *P. aeruginosa* [20,46], and the protein encoded by *pgm* (*cg2800*) possesses 62% identity to the PgmA of *E. coli* [26]. To identify the isoenzyme contributing most to total Pgm activity in *C. glutamicum* cell extracts, we purified the respective isoenzyme from cell extracts using the five-step protocol outlined in the Methods section. In the course of this purification procedure, the fractions with the highest Pgm activities were used for the subsequent purification steps. After separation by SDS/PAGE and CBB staining, two protein bands were detected in the fraction with the highest Pgm activity derived from the final gel filtration step (Figure 3). By MALDI–TOF-MS peptide fingerprinting, the band with a molecular mass of approx. 60 kDa could be assigned to *cg2800*, which was annotated as *pgm*. The second band with a molecular mass of approx, 80 kDA was assigned to orf *cg2523*, which was annotated as *malQ* [18] and probably encodes the 4-α-glucanotransferase of *C. glutamicum*. The analysis of the elution profile from the size exclusion chromatography performed in the course of Pgm purification revealed an oligomeric status. The Pgm protein eluted at a volume between 73.8 and 74.3 ml, corresponding to a molecular mass of approx. 122 kDa. This result indicates that the native Pgm isoenzyme encoded by *pgm* exists as a homo-dimer. Analysis of the activity of the purified Pgm isoenzyme with varying substrate concentrations (0.05–20 mM glc-1-P) revealed a saturation kinetic, with a *K*_M_ of 1.41±0.23 mM and a *V*_max_ of 69.55±5.11 units/mg protein.

To ensure that the isoenzyme encoded by *pgm* indeed contributes most to Pgm activity in *C. glutamicum*, the *cg2800* gene was inactivated by chromosomal insertion of the vector pDrive-IMpgm, resulting in strain *C. glutamicum* IMpgm. In fact, analysis of cell extracts of *C. glutamicum* IMpgm by native PAGE followed by Pgm activity staining revealed one single band, whereas in cell extracts of *C. glutamicum* WT two bands were visible (Figure 2A). The specific activities and kinetic properties of total Pgm in cell extracts of *C. glutamicum* WT and *C. glutamicum* IMpgm cultivated in minimal medium with glucose as sole carbon source were determined using various glc-1-P concentrations (0.05–20 mM). Plotting the data according to the Michaelis-Menten equation revealed saturation kinetics (Figure 2B) with a *K*_M_ of 2.23±0.20 mM and a *V*_max_ of 0.91±0.02 units/mg protein for *C. glutamicum* WT and a *K*_M_ of 1.55±0.36 mM and a *V*_max_ of 0.12±0.01 units/mg protein for *C. glutamicum* IMpgm.

Taken together, our data clearly show that *C. glutamicum* possesses at least two Pgm isoenzymes and that the orf *cg2800* annotated as *pgm* encodes the main Pgm isoenzyme of *C. glutamicum*.

### Characterization of *C. glutamicum* IMpgm

The effects of *pgm* inactivation in *C. glutamicum* on Pgm activity, growth, glycogen content, intracellular glc-1-P levels, cell viability and morphology were studied in the course of growth in minimal medium with either glucose or maltose as a sole carbon source. Independent of the carbon source used for cultivation, total Pgm activities were at least 7-fold lower in cell extracts of *C. glutamicum* IMpgm (for cultivation with glucose 0.08±0.02 units/mg protein or with maltose 0.09±0.01 units/mg protein) than in extracts of *C. glutamicum* WT (for cultivation with glucose 0.66±0.02 units/mg protein or with maltose 0.62±0.08 units/mg protein; activities were measured with 5 mM glc-1-P as substrate). Overexpression of *pgm* using the plasmid pXMJ19-pgm, which carries the *pgm* gene under the control of the IPTG-inducible P*tac* promoter, in *C. glutamicum* IMpgm caused strongly elevated Pgm activities even when compared with activities observed in *C. glutamicum* WT [Pgm activity in *C. glutamicum* IMpgm (pXMJ19-pgm) 1.13±0.19 and 1.31±0.06 units/mg protein for cultivation with glucose or maltose, respectively]. Pgm activities in *C. glutamicum* IMpgm (pXMJ19), which carries the empty plasmid, were about the same as activities observed for *C. glutamicum* IMpgm [Pgm activity in *C. glutamicum* IMpgm (pXMJ19-pgm) 0.07±0.02 and 0.05±0.01 units/mg protein for cultivation with glucose or maltose, respectively].

**Figure 4:**
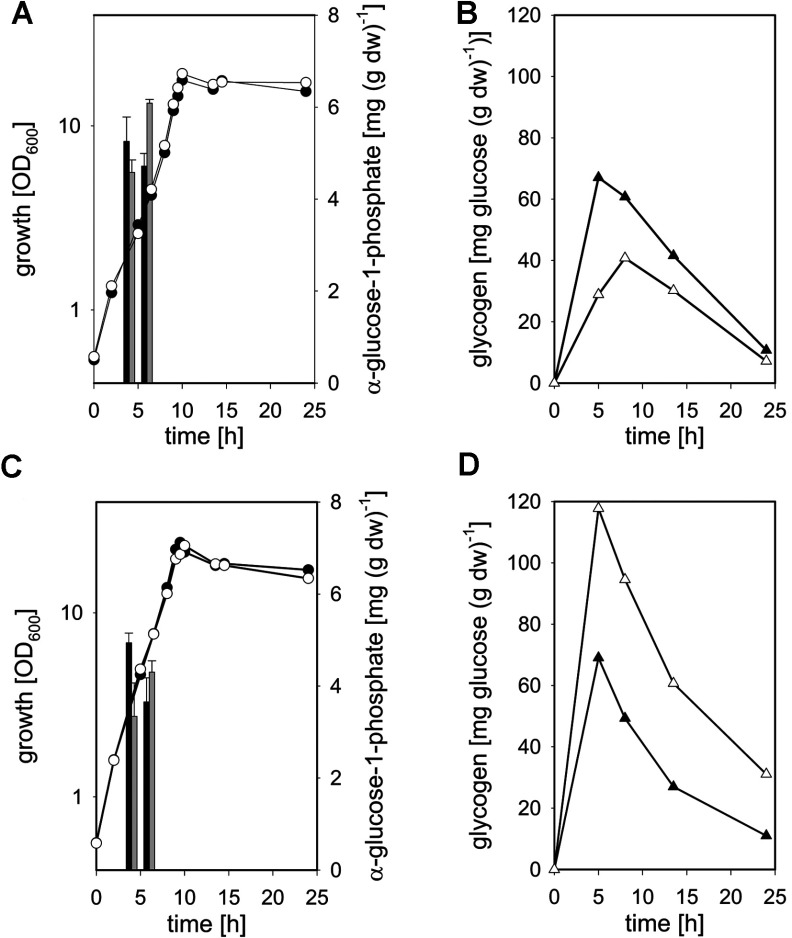
Growth (circles), intracellular glc-1-P levels (bars) and glycogen content (triangles) of *C. glutamicum* WT (filled symbols, black bars) and *C. glutamicum* IMpgm (open symbols, grey bars) in the course of cultivation in minimal medium with 1% (w/v) glucose [(A) growth and glc-1-P levels, (B) glycogen content] or 1% (w/v) maltose [(C) growth and glc-1-P levels, (D) glycogen content] as sole carbon sources Glycogen was determined enzymatically as glucose liberated after amyloglucosidase treatment (two determinations per sample). Three independent cultivations were performed; the S.D.s of the glycogen contents were<10%.

**Figure 5:**
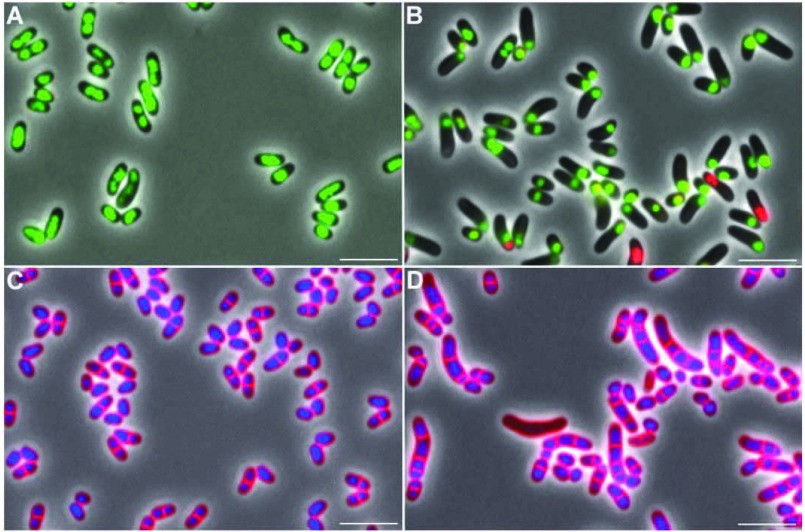
Phenotypes of *C. glutamicum* IMpgm during cultivation in minimal medium with glucose (A, C) or maltose (B, D) Shown are overlays of phase-contrast images with fluorescence images stained with viability stains (**A**, **B**, pictures taken after 7 h of cultivation) or with membrane stain Nile red and DNA stain Hoechst (**C**, **D**, pictures taken after 24 h of cultivation).

As depicted in Figure 4(A), growth of *C. glutamicum* IMpgm in minimal medium with glucose as a carbon source was not affected by the reduced total Pgm activity. Identical growth rates of 0.34±0.05/h were observed for *C. glutamicum* WT and *C. glutamicum* IMpgm and both strains reached comparable final absorbance at 600 nm after 24 h of cultivation (18.8±2.9 and 17.7±0.8, respectively). Also with maltose as a substrate (Figure 4C), no major differences in growth of *C. glutamicum* WT and *C. glutamicum* IMpgm were observed (growth rates of 0.36±0.04 and 0.35±0.02/h and final absorbance at 600 nm of 20.8±2.3 and 21.2±2.4 for WT and IMpgm, respectively). Although the reversible conversion of glc-6-P to glc-1-P catalysed by Pgm was expected to be limited in *C. glutamicum* IMpgm, only minor differences in the intracellular amounts of glc-1-P were measured between WT and the mutant strain (Figures 4A and 4C, bars). However, the glycogen content of the mutant strain was significantly lower during growth with glucose (Figure 4B; maximal glycogen contents of 40.7±8.2 and 67.2±6.4 mg glucose equivalents/g dw were observed for *C. glutamicum* IMpgm and *C. glutamicum* WT, respectively) and significantly higher during cultivation with maltose (Figure 4D, maximal glycogen contents of 117.7±12.1 and 69.0±4.5 mg glucose equivalents/g dw were observed for *C. glutamicum* IMpgm and *C. glutamicum* WT, respectively), when compared with the glycogen levels of *C. glutamicum* WT. No significant changes of cell shape and viability were observed for *C. glutamicum* IMpgm cells in the course of cultivation with glucose (Figures 5A and 5C) and for the WT strain throughout cultivation on both carbon sources. However, the morphology of *C. glutamicum* IMpgm cells drastically changed in the course of cultivations with maltose. As described previously for MalP (maltodextrinphosphorylase)-deficient *C. glutamicum* cells cultivated with maltose [13], most cells of *C. glutamicum* IMpgm cultivated for 24 h with maltose appeared elongated (Figure 5D). The viability assay using the fluorescent nucleic acid stains SYTO9 and propidium iodide indeed revealed that at the mid-exponential growth phase (approx. 7 h after inoculation), *C. glutamicum* IMpgm cells cultivated with maltose were predominantly viable and only slightly enlarged. However, at this time point, the chromosome was very condensed and located at one cell pole (Figure 5B). The viability test also showed that *C. glutamicum* IMpgm cells are for the most part viable after 24 h of cultivation on maltose (results not shown). Furthermore, the phase-contrast microscopic pictures of the viability test showed several stained chromosomes within the enlarged cells after 24 h of cultivation (results not shown). Analysis of these maltose-grown *C. glutamicum* IMpgm cells by fluorescence microscopy using the membrane stain Nile Red and the DNA stain Hoechst indeed showed that these elongated shapes consist of several individual DNA-containing cells divided by membranes (Figure 5D). In contrast, cells of *C. glutamicum* IMpgm cultivated on glucose and stained with Nile Red and Hoechst (Figure 5C), showed the typical morphology of *C. glutamicum* WT cells.

**Figure 6:**
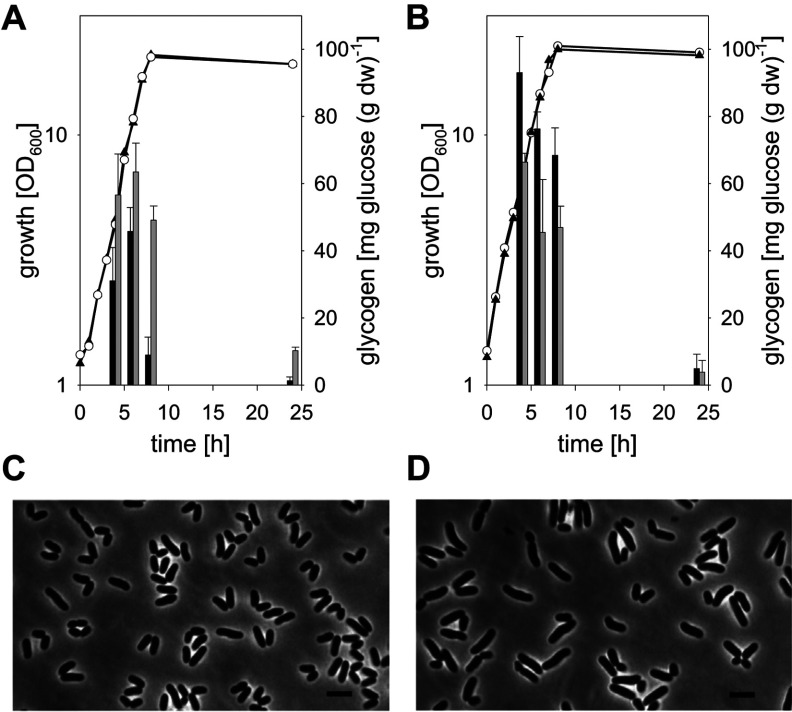
Growth (symbols) and glycogen content (bars) of *C. glutamicum* IMpgm (pXMJ19) (filled triangles, black bars) and *C. glutamicum* IMpgm (pXMJ19-pgm) (open circles, grey bars) in the course of cultivation in minimal medium with 1% (w/v) glucose (A) or 1% (w/v) maltose (B) as sole carbon sources Glycogen was determined enzymatically as glucose liberated after amyloglucosidase treatment (two determinations per sample). Three independent cultivations were performed, data from one representative experiment are shown; the S.D. of the glycogen content was <10%. Phenotypes of *C. glutamicum* IMpgm (pXMJ19-pgm) (**C**) and *C. glutamicum* IMpgm (pXMJ19) (**D**) during cultivation in minimal medium with 1% (w/v) maltose. Shown are phase-contrast images; pictures were taken after 7 h of cultivation.

To rule out the possibility that the effects on glycogen accumulation and cell morphology observed for *C. glutamicum* IMpgm are caused by secondary mutations or polar effects of the integration, we performed complementation studies using the plasmid pXMJ19-pgm. As depicted in Figure 6(A), glycogen accumulation in *C. glutamicum* IMpgm (pXMJ19-pgm) cultivated on glucose was restored to WT levels, whereas in the strain carrying the empty plasmid *C. glutamicum* IMpgm (pXMJ19) the glycogen content remained low (maximal glycogen contents of 63.5±8.6 and 45.7±7.1 mg glucose equivalents/g dw in *C. glutamicum* IMpgm (pXMJ19-pgm) and *C. glutamicum* IMpgm (pXMJ19), respectively). Maltose-grown cells of *C. glutamicum* IMpgm (pXMJ19) accumulated elevated amounts of glycogen (Figure 6B; maximal content of 93.1±8.6 mg glucose equivalents/g dw), as did the strain without plasmid (see above). The maltose-grown cells of *C. glutamicum* IMpgm (pXMJ19-pgm), however, showed glycogen levels comparable to those observed with *C. glutamicum* WT (i.e., maximal glycogen contents of 66.8±2.7 mg glucose equivalents/g dw). Besides, also the drastic effects on cell morphology observed for *C. glutamicum* IMpgm cultivated on maltose were relieved upon plasmid-encoded expression of *pgm.* As shown in Figure 6(C) for maltose-grown *C. glutamicum* IMpgm (pXMJ19-pgm), the typical cell shape of *C. glutamicum* WT cells was observed, whereas the cell shape of *C. glutamicum* IMpgm cells carrying the empty plasmid remained drastically altered. Taken together, these results show that the changes in glycogen content as well as the morphological alterations were caused by the reduced Pgm activity in *C. glutamicum* IMpgm.

## DISCUSSION

We here show that *C. glutamicum* possesses at least two Pgm isoenzymes, the one encoded by *pgm* contributing mostly to the total Pgm activity within the cells. Accordingly, Pgm activity in the *C. glutamicum* strain with inactivated *pgm* was approx. 9-fold lower when compared with *C. glutamicum* WT, but not completely eliminated as reported for *pgm*-deficient (or *pcgA*-deficient) mutant strains of *E. coli*, *B. subtilis, Streptococcus iniae* and *Streptococcus gordonii* [26–28,47]. This result corroborates the finding of a second protein with Pgm activity apart from the *pgm* gene product (Figure 2).

We used the *pgm*-inactivated strain to analyse the effects of limited Pgm activity on morphology, glycogen accumulation and intracellular concentrations of glc-1-P, an intermediate of glycogen metabolism and a precursor for the synthesis of nucleotide sugars and cell wall components such as trehalose and rhamnose [10,12,30,31,48]. Based on the proposed metabolic scheme for maltose and glycogen metabolism in *C. glutamicum* [13], and on the specific activities and kinetic properties of total Pgm in cell extracts of *C. glutamicum* IMpgm (this work), we selected cultivation conditions in which Pgm activity was supposed to limit either the conversion of glc-6-P to glc-1-P required for anabolism (cultivation on glucose) or vice versa the formation of glc-6-P from glc-1-P required for catabolism (cultivation on maltose). We observed drastic changes in cell morphology of *C. glutamicum* IMpgm cells in the course of cultivation with maltose as substrate. Changes of the cell shape and size caused by the inactivation of genes encoding Pgm enzymes have also been reported for *E. coli*, *B. subtilis* and *S. gordonii* [25–28]: whereas cells of a *pgm* deficient *E. coli* strain are approx. 70% shorter but also slightly wider than cells of the parental strain [26], cell diameters of *pgm*-deficient strains of both *S. iniae* and *S. gordonii* are increased [28,47]. For *pgm*-deficient *B. subtilis* strains different cell morphologies were observed. Lazarevic et al. [27] reported that *B. subtilis pgm* mutants adopt a spherical shape; however, the *B. subtilis* cells of the *pgm*-mutant described by Weart et al. [25] were shorter as cells of the parental strain but still rod-shaped. The lack of Pgm activity in these bacteria has been proposed to limit the availability of the common precursor glc-1-P and thereby to affect the synthesis of cell wall components such as the lipoteichoic acids in *B. subtilis* and *Staphylococcus aureus* [25,49], the LPS (lipopolysaccharides) of the outer membrane in *Agrobacterium tumefaciens* and *Brucella abortus* [50,51], and the capsular polysaccharides in *Streptococcus pneumonia* [52].

Also for *C. glutamicum* limited glc-1-P availability has been suggested to cause the drastically altered cell morphology and decreased viability in maltose-grown cells of *C. glutamicum* ΔmalP [13]. However, we exclusively observed elongated cells during cultivations of *C. glutamicum* IMpgm on maltose, cultivation conditions initially supposed to favour accumulation of glc-1-P. *Albeit* we did not observe significant alterations of the intracellular glc-1-P levels between *C. glutamicum* WT and *C. glutamicum* IMpgm in the course of cultivations with both glucose and maltose as a carbon source, the changes in the glycogen content in *C. glutamicum* IMpgm reflected the initially conceived changes in the availability of its precursor glc-1-P. As expected, the glycogen content in the *pgm* mutant strain was reduced in cells cultivated on glucose and increased in cells cultivated on maltose. Since glc-1-P is both a precursor and a degradation product of glycogen metabolism, it can be speculated that a constant level of glc-1-P in the course of cultivation is maintained by coordination of glycogen synthesis and degradation in *C. glutamicum.* Such a glc-1-P homoeostasis reflects the proposed role of glycogen as a carbon capacitor in *Corynebacterianeae* [22,53]. The concept of glycogen as carbon capacitor was initially proposed for *Mycobacterium smegmatis* as both excessive synthesis and recycling of glycogen were observed in the course of the exponential growth phase [53], an observation that does not fit to the generally accepted role of glycogen in non-sporulating bacteria as a long-term energy reserve required for the survival in substrate-limited environments [11,12,17]. For the latter function (i.e., slow degradation of glycogen in the course of the stationary growth phase), the energy necessary for maintenance in the absence of extracellular substrates is provided [11]. *C. glutamicum*, however, degrades the majority of the accumulated glycogen before the onset of the stationary growth phase and, moreover, survival of a glycogen synthesis-deficient strain, *C. glutamicum* IMglgC, was not reduced upon prolonged incubation when compared with the parental strain [8]. Since the reactions for glycogen synthesis and degradation take place simultaneously in exponentially growing *C. glutamicum* cultures [22], we adapted the concept of glycogen as a carbon capacitor also as a model for *C. glutamicum*. This function of glycogen as a carbon capacitor during growth is also supported by the recent findings of Koch-Koerfges et al. [54]. These authors found that exponentially growing cells of *C. glutamicum* show endogenous respiration in the absence of an external energy source, proceeding at a rate of approx. 50% of the respiration rate in the presence of glucose. In contrast, the endogenous respiration was significantly lower in cells starved for 3 h before the measurement. The first observation – high rate of endogenous respiration in the absence of external substrates – reflects the ability of *C. glutamicum* to degrade glycogen to level fluctuations in substrate availability whereas the second observation – low endogenous respiration in starved cells – reflects that glycogen is not a long-term storage compound in *C. glutamicum*. Taken further into account (i) the importance of the cell wall component and compatible solute trehalose for growth and viability of *Corynebacterianeae* [30,55,56], (ii) the abundance of interconnections between the pathways for glycogen metabolism and synthesis of trehalose in this group of bacteria (reviewed in [57,58]) and (iii) the interplay between glycogen metabolism and homoeostasis of glc-1-P (the precursor for both glycogen and trehalose synthesis) as shown here, the concept of glycogen as a carbon capacitor to metabolic (carbon) fluctuations seems quite reasonable. However, glycogen synthesis-deficient *C. glutamicum* strains (e.g., *C. glutamicum* IMglgC and *C: glutamicum* IMglgB) showed identical growth rates and final absorbance at 600 nm as the parental strain when cultivated in a CgC minimal medium with glucose as a sole source of carbon and energy [8,14]. The lack of a growth phenotype for glycogen synthesis-deficient *C. glutamicum* strains might be explained by the nearly constant and more or less optimal conditions in the course of the cultivations (i.e., shake flask experiments using well pH-buffered CgC minimal medium or pH-, pO_2_- and temperature-controlled batch-fermentations in small bioreactors). The importance of glycogen (metabolism) for growth and fitness of *C. glutamicum* might be observed in the course of large-scale industrial cultivations, when abiotic parameters repeatedly change.

Our results indicate that a decreased intracellular availability of glc-1-P and thus, reduced levels of cell wall components are not responsible for the observed cell elongations in *C. glutamicum* strains. As morphology of both *C. glutamicum* ΔmalP and *C. glutamicum* IMpgm were changed in the course of cultivation with maltose and unaffected during cultivation with glucose, it seems reasonable that the accumulation of intermediates of the maltose metabolism, e.g., maltodextrins or the drastically increased accumulation of glycogen induce these morphological changes in *C. glutamicum* cells with abolished MalP or reduced Pgm activity.
